# Drug-Resistant Tuberculosis Transmission and Resistance Amplification within Families

**DOI:** 10.3201/eid1808.111650

**Published:** 2012-08

**Authors:** James A. Seddon, Rob M. Warren, Donald A. Enarson, Nulda Beyers, H. Simon Schaaf

**Affiliations:** London School of Hygiene and Tropical Medicine, London, UK (J.A. Seddon);; Stellenbosch University, Tygerberg, South Africa (J.A. Seddon, R.M. Warren, N. Beyers, H.S. Schaaf);; International Union Against Tuberculosis and Lung Disease, Paris, France (D.A, Enarson);; and Tygerberg Children’s Hospital, Tygerberg (H.S. Schaaf)

**Keywords:** Tuberculosis, child, drug resistance, Mycobacterium tuberculosis, tuberculosis and other mycobacteria

## Abstract

Drug-resistant tuberculosis is caused by transmission of resistant strains of *Mycobacterium tuberculosis* and by acquisition of resistance through inadequate treatment. We investigated the clinical and molecular features of the disease in 2 families after drug-resistant tuberculosis was identified in 2 children. The findings demonstrate the potential for resistance to be transmitted and amplified within families.

The devastating effects of extensively drug-resistant tuberculosis (XDR TB) gained international attention after the 2006 outbreak in Tugela Ferry, South Africa. The evolution of the epidemic is the result of transmission of resistant strains and strain acquisition of resistance through inadequate treatment (*1*). Multidrug-resistant (MDR) TB is disease caused by *Mycobacterium tuberculosis* that is resistant to isoniazid and rifampin, and XDR TB is disease caused by *M. tuberculosis* that is additionally resistant to a fluoroquinolone and an injectable second-line anti-TB drug. Because children usually have transmitted resistance (*2*), they can be seen as the end of a sequence of transmission events. We describe investigations of 2 families after the identification of children with drug-resistant TB in terms of clinical features and molecular characteristics of the isolates.

## The Study

This investigation was conducted in a suburban community of Cape Town, South Africa, where TB incidence was 978/100,000 population in 2009 (Health Systems Trust). Since 1994, microbiological samples from all patients treated for TB in this area have been sent to the research laboratory at Tygerberg Hospital, Stellenbosch University. From 2008 through 2010, two children from this community received a diagnosis of MDR TB.

Information was obtained from several sources to document the sequence of events that culminated in the development of MDR TB in each child. A home visit was made, and the family was interviewed after written informed consent was obtained. Family members were included if they either lived with or spent substantial amount of time with the child (*3*). Information on TB diagnosis, treatment, and outcome was obtained at interview. If a family member was identified as having had TB, family contacts of that person were included. Searches for case notes for those included were made at the local clinic, the academic hospitals, and the regional TB hospital responsible for drug-resistant TB management. Also, the local clinic TB register was consulted. The investigation was approved by the Stellenbosch University Ethics Committee.

Sputum samples from the 2 families were identified, and isolates were genotyped by spoligotyping (*4*) and IS*6110* DNA fingerprinting (*5*). Strains were identified according to distinct IS*6110* banding patterns by using Gelcompar II (Applied Maths, Sint-Martens-Latem, Belgium) or characteristic spoligotype pattern (*6*). Mutations conferring resistance to isoniazid, rifampin, ethambutol, pyrazinamide, ofloxacin, and amikacin were determined by DNA sequencing of the *inhA* promoter, *katG*, *rpoB*, *embB, pncA*, *gyrA,* and *rrs* genes, respectively (*7*).

A 19-month-old girl (A3) received a diagnosis of TB in March 2008 after a 6-month course of preventive therapy with isoniazid. She was brought for assessment with a 2-weeek history of cough, respiratory distress, and fever. She had contact with a patient with pre–XDR TB (MDR TB resistant to either a fluoroquinolone or a second-line injectable drug), and therefore the following antimicrobial drugs were administered: capreomycin, ethionamide, ethambutol, *para*-aminosalicylic acid, terizidone, clarithromycin, and high-dose isoniazid. Gastric aspirate samples were sent to the National Health Laboratory Service; *M. tuberculosis* grew in culture and was resistant to rifampin, isoniazid, and ofloxacin and susceptible to amikacin and ethionamide. She received treatment for 18 months from the time of her first negative culture (the first 6 months included the injectable medication) and recovered.

**Figure 1:**
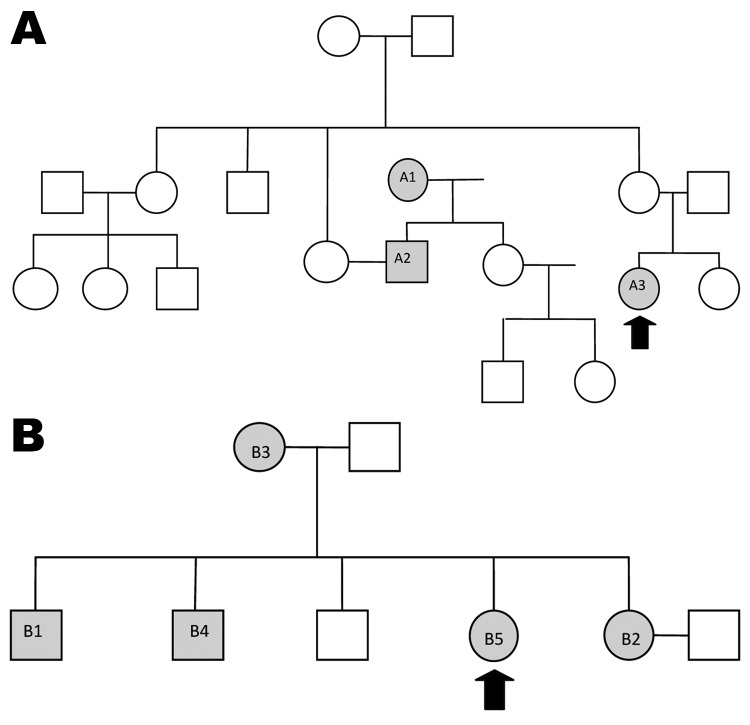
Patients with drug-resistant tuberculosis in families 1 (A) and 2 (B), South Africa, 2008–2010. Gray shading indicates person identified with tuberculosis; arrows indicate child index case-patients; circles indicate female family members; squares indicate male family members.

**Figure 2:**
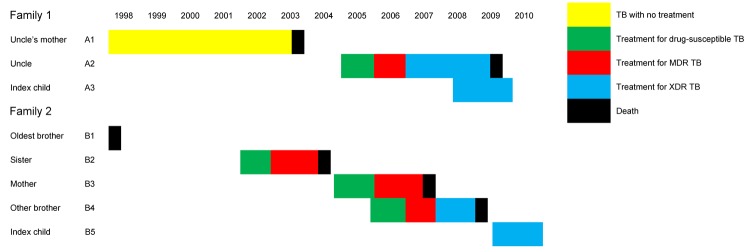
Chronology of tuberculosis treatment and outcomes for 2 families with drug-resistant tuberculosis (TB), South Africa, 2008–2010. MDR TB, multidrug-resistant TB; XDR TB, extensively drug-resistant TB.

Patient 1’s family consisted of 18 persons (Figure 1). The husband of her aunt (A2) had drug-resistant TB. He cared for the girl on a daily basis. He had received treatment initially for drug-susceptible TB; this was changed to MDR TB therapy when resistance to rifampin and isoniazid was determined and then to XDR TB treatment when resistance to second-line drugs was discovered. He subsequently died. His mother (A1) had repeatedly dropped out of treatment, and drug-resistant TB was finally diagnosed in 1998. She refused further treatment and died in 2003. The clinical chronology is shown in Figure 2; molecular details regarding the samples analyzed are shown in the Table.

**Table:** Gene sequencing, IS*6110* DNA fingerprinting, and genotype results for isolates from members of 2 families with drug-resistant tuberculosis, South Africa, 2008–2010*

Family and family member	ID no.	Gene†							IS*6110* cluster no.
		*rpoB*	*inhA*	*katG*	*embB*	*gyrA*	*pncA*	*rrs* 1401	
		R	H	H	E	F	Z	A	
Family 1									
Uncle’s mother	A1	531, TCG→TTG	WT	315, AGC→ACC	306, ATG→ATA	90, GCG→GTG	160, ACA→GCA; 100, ACC→ATC	WT	213
Uncle	A2	531, TCG→TTG	WT	315, AGC→ACC	306, ATG→ATA	90, GCG→GTG	160, ACA→GCA; 100, ACC→ATC	WT	213
Index child	A3	531, TCG→TTG	WT	315, AGC→ACC	306, ATG→ATA	90, GCG→GTG	160, ACA→GCA; 100, ACC→ATC	WT	§
Family 2									
Oldest brother‡	B1								
Sister	B2	531, TCG→TTG	WT	315, AGC→ACC	306, ATG→ATA	90, GCG→GTG	160, ACA→GCA; 100, ACC→ATC	WT	213
Mother	B3	531, TCG→TTG	WT	315, AGC→ACC	306, ATG→ATA	90, GCG→GTG	160, ACA→GCA; 100, ACC→ATC	1401, ACG→GCG	213
Other brother	B4	531, TCG→TTG	WT	315, AGC→ACC	306, ATG→ATA	90, GCG→GTG	160, ACA→GCA; 100, ACC→ATC	1401, ACG→GCG	213
Index child	B5	531, TCG→TTG	WT	315, AGC→ACC	306, ATG→ATA	90 GCG→GTG	160, ACA→GCA; 100, ACC→ATC	1401, ACG→GCG	§

*All isolates were *Mycobacteria tuberculosis* Beijing genotype. The earliest sample available for each patient is shown; in all instances in which >1 sample was available for a patient, all samples demonstrated identical gene sequence and strain type results. ID, identification; R, rifampin; H, isoniazid; E, ethambutol; F, fluoroquinolones; Z, pyrazinamide; A, aminoglycosides, WT, wild type. †Numbers indicate specific mutations, which are shown. ‡Tuberculosis developed and patient died before systematic sample collection and storage. No culture or drug susceptibility testing was requested for sample. §Only spoligotyping performed because isolates repeatedly lost viability on culture.

A 13-year-old girl (B5) was identified in April 2009 as a contact of multiple family members with XDR TB. She was asymptomatic, but a chest radiograph showed abnormalities. A regimen was begun of capreomycin, ethionamide, pyrazinamide, terizidone, *para*-aminosalicylic acid, co-amoxicillin/clavulanic acid, clarithromycin, linezolid, and high-dose isoniazid. *M. tuberculosis,* cultured from a sputum sample, was resistant to isoniazid, rifampin, ethambutol, ofloxacin, and amikacin. Capreomycin was given for 6 months, and she received treatment for 18 months in total. The condition was cured.

Patient 2’s family is depicted in Figure 1. The eldest brother (B1) had been in prison, and TB developed soon after his release in 1998. First-line treatment was begun, but he died soon afterward. TB then developed in his sister (B2), mother (B3), and brother (B4). All were given first-line therapy, which was changed, when resistance profiles became available, to the regimen for MDR TB and, for the brother, to the regimen for XDR TB. All 3 patients died. A chronology is shown in Figure 2; molecular details regarding the samples are provided in the Table.

## Conclusions

In family 1, the uncle’s mother (A1) had pre–XDR TB and probably transmitted it to her son (A2). He likely transmitted it to his niece (A3). Strains for all 3 were identical. In family 2, whether the oldest brother (B1) had drug-resistant TB is unknown. His sister (B2) had pre–XDR TB; then, in sequence, XDR TB developed in her mother (B3), brother (B4), and sister (B5), caused by a strain identical to hers. This investigation, therefore, demonstrates the potential for resistance to be transmitted and amplified within families.

Other than the 2 index case-patients (A3 and B5), all were initially given first-line therapy and received treatment until drug susceptibility test (DST) results became available, often despite a drug-resistant contact being known. Local policy is to diagnose TB solely from sputum smear in new patients who have no risk factors for drug resistance. Retreatment patients and those at risk for resistance have strains tested for drug susceptibility to rifampin and isoniazid. If MDR TB is diagnosed, DST to second-line drugs is then performed. Giving inadequate regimens not only leads to more advanced disease until effective treatment is initiated but also risks amplifying resistance (*8**,**9*). For a patient with TB symptoms who is in contact with a drug-resistant TB patient, it is essential to obtain microbiological samples and then start treating the disease according to the DST results for the source case. If a less-resistant organism is grown, treatment can be changed. In the context of multiple possible TB sources, deciding on treatment is challenging. Consideration must be given to the infectiousness of potential sources as well as the intensity, frequency, and duration of exposures. Local policy is to carry out household contact tracing for drug-resistant TB patients. Although this tracing occurs infrequently, we demonstrate the value of careful investigation of contacts to identify those who may have subclinical disease that could be treated at an early stage. Given the social interactions, chronology of illness, and the results of mycobacterial cultures, the transmission sequence in these cases likely occurred as described. However, in both clusters, the strain identified was the predominant local strain and is a potential confounder to the transmission lines suggested.

With the rollout of rapid, genotypic diagnostic tests (*10*), which ultimately should be extended to all persons suspected of having TB, more drug-resistant TB may be diagnosed correctly and earlier. If this leads to prompt, appropriate treatment, further transmission and amplification of resistance could be reduced. For XDR TB treatment, drug options that are not only new to the patient but also new to the community must be available. The use of linezolid and other novel drugs will become crucial in the management of an evolving drug-resistant TB epidemic.
